# Supplementary material for: Biomonitoring of Airborne Microplastic Deposition in Semi-Natural and Rural Sites Using the Moss Hypnum cupressiforme
Source: Plants (Basel). 2023 Feb 21;12(5):977. doi: 10.3390/plants12050977 (PMC10005416; doi:10.3390/plants12050977)
Supplement: Supplementary file 1 [file plants-12-00977-s001.zip › Table S1.pdf]

Table S1. Microplastic distribution (%) divided into size classes from 0 to 5 mm

Classes* mm	MTF	MTV	ARP	PTR	MTB	MRC	MTS
**<0.2**	4	7	6	6	4	6	4
**0.2-0.4**	5	6	6	6	6	6	10
**0.4-0.6**	5	11	10	9	8	6	8
**0.6-0.8**	10	14	10	9	12	12	11
**0.8-1.0**	8	8	10	10	13	9	7
**1.0-1.2**	7	7	10	9	15	10	7
**1.2-1.4**	6	6	9	8	8	12	8
**1.4-1.6**	5	8	7	10	11	12	5
**1.6-1.8**	5	11	6	8	9	7	9
**1.8-2.0**	7	3	4	6	6	4	6
**2.0-2.2**	8	8	8	4	5	6	6
**2.2-2.4**	5	3	2	3	1	3	9
**2.4-2.6**	5	4	4	2	2	3	2
**2.6-2.8**	5	1	2	1	0	1	4
**2.8-3.0**	4	1	1	2	0	2	2
**3.0-3.2**	2	1	2	3	0	1	1
**3.2-3.4**	0	0	0	1	0	0	1
**3.4-3.6**	1	1	0	1	0	0	0
**3.6-3.8**	1	0	0	0	0	0	0
**3.8-4.0**	3	0	0	0	0	0	0
**4.0-4.2**	0	0	0	1	0	0	1
**4.2-4.4**	0	0	0	0	0	0	0
**4.4-4.6**	1	0	0	0	0	0	0
**4.6-4.8**	1	0	0	0	0	0	0
**4.8-5.0**	0	0	0	0	0	0	0

*Each distribution class have Lower value ≤N° of MPs< Higher value
