# Supplementary figures and images for: Biomonitoring of Airborne Microplastic Deposition in Semi-Natural and Rural Sites Using the Moss Hypnum cupressiforme
Source: Plants (Basel). 2023 Feb 21;12(5):977. doi: 10.3390/plants12050977 (PMC10005416; doi:10.3390/plants12050977)

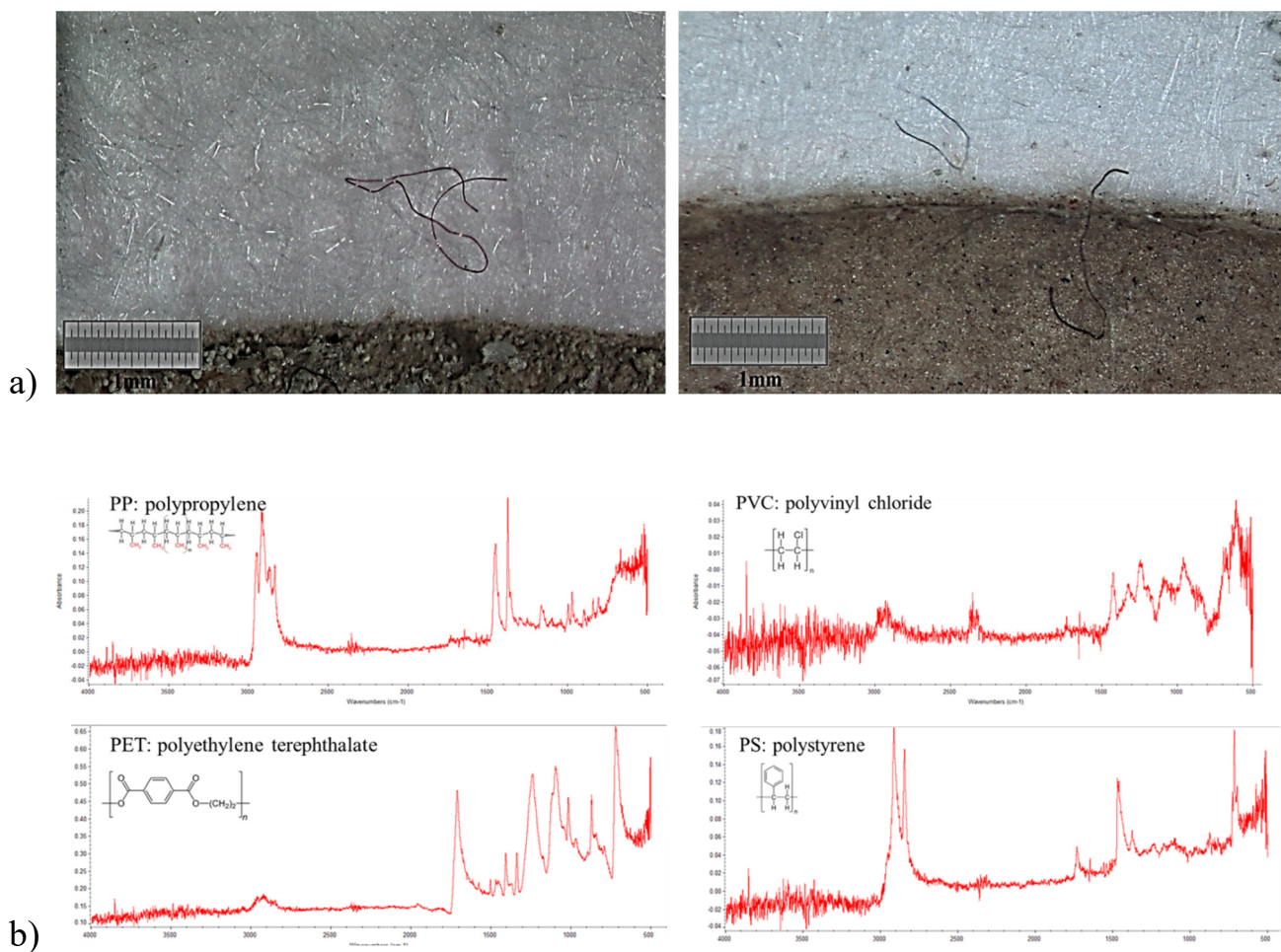

Supplement: Supplementary file 1 [file plants-12-00977-s001.zip › Figure S1.pdf]
